# SP140 inhibitor suppressing TRIM22 expression regulates glioma progress through PI3K/AKT signaling pathway

**DOI:** 10.1002/brb3.3465

**Published:** 2024-03-11

**Authors:** Xiang Li, Guangzhao Li, Longyuan Li, Bixi Gao, Xiaowang Niu, Yunjiang Wang, Zhong Wang

**Affiliations:** ^1^ Department of Neurosurgery The First Affiliated Hospital of Soochow University Suzhou China; ^2^ Department of Neurosurgery Xinghua People's Hospital Xinghua China; ^3^ Department of Neurosurgery Hefei First People's Hospital Hefei China; ^4^ Department of Neurosurgery The Affiliated Suqian Hospital of Xuzhou Medical University Suqian China; ^5^ Department of Neurosurgery Yancheng Third People's Hospital Yancheng China

**Keywords:** glioma, nomogram, prognosis, SP family, TRIM22

## Abstract

**Background:**

SP gene family, consisting of SP100, SP110, SP140, and SP140L, has been implicated in the initiation and advancement of numerous malignancies. Nevertheless, their clinical significance in glioma remains incompletely understood.

**Method:**

Expression levels and prognostic significance of SP family members were evaluated in the TCGA and CGGA datasets. Multifactorial analysis was used to identify SP gene family members that can independently impact the prognosis of glioma patients. A SP140‐based predictive risk model/nomogram was developed in TCGA dataset and validated in CGGA dataset. The model's performance was evaluated through receiver operating characteristic (ROC) curves, calibration plots, and decision curve analyses. Phenotypic associations of SP140 and TRIM22 were examined through CancerSEA and TIMER. The effect of SP140 inhibitor in glioma progress and TRIM22/PI3K/AKT signaling pathway was confirmed in U251/U87 glioma cells.

**Results:**

The SP family members exhibited elevated expression in gliomas and were negatively correlated with prognosis. SP140 emerged as an independent prognostic factor, and a SP140‐based nomogram/predictive risk model demonstrated high accuracy. SP140 inhibitor, GSK761, lead to the suppression of TRIM22 expression and the PI3K/AKT signaling pathway. GSK761 also restrain glioma proliferation, migration, and invasion. Furthermore, SP140 and TRIM22 coexpressed in glioma cells with high level of vascular proliferation, TRIM22 is closely associated with the immune cell infiltration.

**Conclusion:**

SP140‐based nomogram proved to be a practical tool for predicting the survival of glioma patients. SP140 inhibitor could suppress glioma progress via TRIM22/PI3K/AKT signaling pathway.

## Graphical abstract

Flowchart of this study. Gene changes, prognostic model, and function assessment of SP100 gene families in glioma.

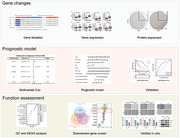

## 1 INTRODUCTION

Glioma is indeed a highly aggressive and deadly type of brain tumor in adults (Laws et al., 2003). Despite the development of new treatment strategies involving surgical resection combined with chemoradiotherapy, the prognosis for glioma patients remains bleak, with an average survival period of only approximately 14.6 months (Hu et al., 2018; Jiang et al., 2021). The rapid progression of the disease, coupled with the considerable heterogeneity of gliomas, presents significant challenges in predicting patient outcomes (Hu et al., 2018; Jiang et al., 2021). Molecular markers have revealed the complexity of glioma prognosis, highlighting that a single parameter cannot accurately predict the course of the tumor. Therefore, the identification of novel and effective prognostic models and drug targets is an urgent and critical task. This not only has implications for the management of glioma but also for the discovery of potential therapeutic drugs.

The Speckled Protein (SP) family consists of four distinct members: SP100, SP110, SP140, and SP140L. These members share common features, which are characterized by the presence of two specific domains: the HSR (homogeneously staining region) domain and the SAND (Sp100, AIRE‐1, NucP41/75, and DEAF‐1) domain (Mehta et al., 2017). These common features, including the HSR and SAND domains, suggest that the members of the SP family may have similar functions or interact with similar molecular pathways. These proteins are implicated in various human diseases, including cancer, immune disorders, autoimmune diseases, and viral infections (Fraschilla & Jeffrey, 2020). In immune disorders, mutations in SP110 have been linked to immunodeficiency disorders, making individuals more susceptible to mycobacterial infections (Lei et al., 2012). Additionally, SP140 alterations have been associated with autoimmune diseases like primary biliary cirrhosis (PBC), where the immune system mistakenly attacks the body's own tissues (Granito et al., 2010). In cancer, SP family proteins, such as SP100, have been associated with malignancies like acute promyelocytic leukemia (APL) and breast cancer. Altered expression or mutations in these proteins can influence gene regulation and potentially contribute to tumor development (Seeler et al., 1998; Yordy et al., 2004). A previous study demonstrated a significant association between high SP family member expression in pancreatic adenocarcinoma (PAAD) and unfavorable clinicopathological characteristics as well as a poor prognosis for patients with PAAD (Duan et al., 2023). SP110 and SP140 showed high rates of nonsynonymous mutations in head and neck squamous cell carcinoma (Chang et al., 2020). Besides, SP140 is highly expressed in tumor‐associated macrophages across head and neck squamous cell carcinoma, and higher expression of SP140 in the tumors was associated with higher tumor mutation burden, improved survival, and a favorable response to immunotherapy (Tanagala et al., 2022). In patients with sleep disordered breathing and excessive daytime sleepiness, SP140 promoter methylation (−194 CpG site) was increased, and SP140 protein levels were decreased (Chen et al., 2016). SP140 has been implicated by genome‐wide association studies (GWAS) as risk factor in multiple sclerosis (International Multiple Sclerosis Genetics, Wellcome Trust Case Control et al., 2011). These studies suggest that SP140 may also play an important role in the nervous system, especially in neuron or glia. In central nervous system tumor, SP100 is relatively highly expressed in meningiomas (Held‐Feindt et al., 2011). Additionally, in gliomas, SOX2 can lead to a worse prognosis by downregulating PML and SP100 (Wen et al., 2023). This suggested that SP family may play a significant role in gliomas. However, the roles of other members of the SP family in gliomas remain largely unknown. There was a lack of comprehensive research on the involvement of the SP family in gliomas.

In our study, we conducted a comprehensive investigation into the potential roles and underlying mechanisms of SP family members in glioma development and progression. We utilized public databases and employed various bioinformatics analysis techniques to achieve this. Additionally, we conducted survival analysis, using Kaplan–Meier and Cox regression analyses, to identify independent clinicopathologic factors. Subsequently, we constructed and validated a novel nomogram and prognostic risk model using glioma cohorts from TCGA and CGGA databases. Finally, we delved into the potential mechanisms through which SP140 regulates glioma proliferation and invasion. These findings offer valuable insights that may unveil prospective therapeutic targets for addressing this challenging disease.

## 2 METHOD AND MATERIALS

### 2.1 Data collection

The mRNA sequencing data for glioma we collected in this study were primary glioma samples from the Cancer Genome Atlas (TCGA) and Chinese Glioma Genome Atlas (CGGA) databases. The mRNA sequencing data of glioma samples and clinical patient information were downloaded and sorted from TCGA (https://portal.gdc.cancer.gov/) and CGGA (http://www.cgga.org.cn/). In addition, the mRNA expression data of normal brain tissue samples were downloaded from GTEX, and the expression of SP family genes was extracted and analyzed the difference with the expression from TCGA and CGGA. All data were standardized and analyzed by R (4.3.2).

### 2.2 Cell culture

Human astrocyte HA and human glioma cell lines U87, U118, and U251 were obtained from the Shanghai Institutes for Biological Science and subjected to short tandem repeat (STR) DNA profiling for authentication. These cell lines were cultured in a humidified incubator at 37°C with 5% CO2, using Dulbecco's Modified Eagle Medium (DMEM) supplemented with 10% fetal bovine serum (FBS) sourced from Shanghai Zhongqiao Xinzhou Biotechnology Co., Ltd, China. The culture medium was refreshed daily.

### 2.3 Cell viability assay (CCK‐8 assay)

GSK761 (0.1 µM) was introduced into a 96‐well plate containing 100 µL of cell culture medium. Following incubation for 1, 2, 3, and 4 days, 10 µL of CCK‐8 reagent obtained from Beyotime, China, was added to each well. Subsequently, the plate was incubated in a constant temperature incubator at 37°C for 2 h. To evaluate cell viability, the variance in optical density values at 450 nm was measured using an enzyme‐based assay.

### 2.4 Genetic alteration analysis of SP family

cBioPortal (http://www.cbioportal.org/) is a genetic alteration analysis site based on the TCGA database. We used cBioPortal, including 514 low‐grade gliomas (LGG) (TCGA, Pancancer atlas) and 577 glioblastoma (GBM) (TCGA, cell 2013) to analyze different genetic alteration types of the SP family genes. In addition, we analyzed all genetic alteration types in these samples simultaneously.

### 2.5 Human protein atlas

We employed the Human Protein Atlas (HPA), a comprehensive resource providing immunocytochemistry‐based protein expression data across 20 different cancer types, to explore variations in the expression patterns of model genes between glioma and normal brain tissues. This analysis was carried out utilizing the online tools accessible on the HPA website (https://www.proteinatlas.org) (Asplund et al., 2012).

### 2.6 Survival analysis, model construction, and validation

We conducted an extensive analysis of the relationship between SP family genes and clinical outcomes, including overall survival rate and survival time in glioma samples from both the TCGA and CGGA databases, utilizing the Kaplan–Meier method and log‐rank test. Additionally, we employed univariate and multivariate Cox regression analyses to investigate the connection between SP family expression and various clinical parameters, such as age, sex, IDH mutation status, WHO classification, and 1p/19q deletion. To integrate gene expression data with the aforementioned clinical information for visualization, we utilized the RMS software package in R (version 4.3.2) to create a nomogram. This nomogram serves as a predictive tool for assessing glioma prognosis at 1, 3, and 5 years postdiagnosis. The predictive risk score model, constructed via multivariate Cox analysis, incorporated independent prognostic factors. The calculation for the predictive risk score is as follows:

$$\mathrm{Risk}\;\mathrm{score}\;=\;\left(0.0137\times \mathrm{age}\right)+\left(-0.8248\times \mathrm{IDH}\;\mathrm{mutation}\right)+\left(0.9180\times \mathrm{Grade}\;\mathrm{III}\;\mathrm{or}\;1.4916\times \mathrm{Grade}\;\mathrm{IV}\right)+\left(0.1071\times \mathrm{SP}140\;\mathrm{expression}\right).$$

We employed time‐dependent receiver operating curves (ROC), calibration curves, and decision curve analysis to validate the accuracy of the predictive risk scores. To assess the generalizability of the risk scores, we tested them on gliomas in TCGA database and CGGA database.

In all our analyses, statistical significance was defined as *p* < .05. We conducted these analyses using analysis and visualization tools available on the online bioinformatics analysis website (https://www.xiantao.love/) and R (version 4.3.2).

### 2.7 Identification of potential downstream gene regulated by SP140

To identify potential downstream proteins that may function in conjunction with SP140, we performed differential expression analysis on mRNA transcripts of all coding proteins between the high and low expression of SP140 groups. Then we conducted coexpression analysis of SP140. We used a threshold of Log Fold Change (LogFC) > 1 and *p* < .001 to identify mRNAs with differential expression. Subsequently, we conducted enrichment analysis using GO (www.geneontology.org) and KEGG (www.genome.jp/kegg) databases to elucidate any potential differences in biological or cellular functions that may exist between the high and low expression of SP140 groups. Genemania (www.genemania.org) database was used to identified the interaction proteins of SP family. Friends analysis method assesses functional correlations among different genes within a pathway, indicating that a gene is more likely to be expressed and widely used for identifying key genes if it interacts with other genes within the same pathway. To identify crucial downstream gene of SP140, the R software package GOSemSim (Yu, 2020) was employed to calculate the functional relatedness among the intersection genes associated with SP140.

### 2.8 Real‐time PCR

Total RNA was extracted using RNAiso Plus (Takara, Dalian), and cDNA synthesis was conducted with the RevertAid First Strand cDNA Synthesis Kit (Thermo Fisher Scientific, Waltham, MA, USA), following the manufacturer's guidelines. β‐actin served as the internal reference, and real‐time quantitative polymerase chain reaction (RT‐qPCR) was carried out using a SYBR Green‐based fluorescence quantification approach. The primers involved in this study were β‐actin‐F: CATGTACGTTGCTATCCAGGC, β‐actin‐R: CTCCTTAATGTCACGCACGAT, TRIM22‐F: CTGTCCTGTGTGTCAGACCAG, TRIM22‐R: TGTGGGCTCATCTTGACCTCT. The obtained results were analyzed utilizing the 2^−∆∆Ct^ method to determine the relative fold change in RNA expression.

### 2.9 Western blot

Cells treated as specified were harvested and then lysed using Radio Immunoprecipitation Assay (RIPA) lysis buffer, supplemented with protease and phosphatase inhibitors. The protein lysates were subsequently separated on either 10% or 12% SDS‐PAGE gels and transferred onto nitrocellulose filter membranes. Primary antibodies were applied to target the specific proteins overnight at 4°C, followed by a 1‐h incubation with secondary antibodies at room temperature. To visualize protein bands, an enhanced chemiluminescence solution (Thermo Fisher Scientific, Waltham, Massachusetts, USA) was added to the membranes, and the protein bands were detected using a luminescent image analyzer (Clinx ChemiScope5300, Shanghai, China). The intensity of the bands was quantified using Image J software (NIH, USA). The primary antibodies used in this study were anti‐PI3K antibody (67121‐1‐Ig, Proteintech), anti‐AKT antibody (60203‐2‐Ig, Proteintech), anti‐pho‐AKT antibody (80455‐1‐RR, Proteintech), anti‐TRIM22 antibody (ab68071, Abcam).

### 2.10 Transwell invasion assay

Following the manufacturer's instructions, a Matrigel‐coated transwell chamber from ThermoFisher was employed to assess the invasive capability of U87 and U251 cells. The transwell chamber, sourced from Corning, had an 8 µm pore size. For the experiment, 2 × 10^4^ U87 or U251 cells were suspended in 200 µL of Neurobasal‐A medium (Gibco, USA), and this cell suspension was added to the upper compartment of the transwell. The lower compartment was filled with 800 µL of culture medium supplemented with 5% fetal bovine serum (FBS). Following a 24‐h incubation period, cells on the lower surface were fixed with methanol for 15 min. Subsequently, these cells were stained with a 1% crystal violet solution for 30 min and quantified using ImageJ software (version 1.8.0).

### 2.11 Wound‐healing assay

U87 and U251 cells (5 × 10^5^ cells/well) were plated in a 6‐well plate and allowed to incubate overnight at 37°C with 5% CO2. The following day, a horizontal scratch was made across the cell monolayer using the tip of a 10 µL pipette. To remove any dislodged cells, the cells were then thoroughly washed three times with PBS. Afterward, serum‐free culture medium was added to the wells, and the cells were maintained in a 37°C incubator with 5% CO_2_. Time‐lapse images were captured at specific time intervals, including 0 and 24 h, using an inverted microscope.

### 2.12 Immune infiltration and immune checkpoint analysis

We investigated the association between the target gene's expression and the tumor immune microenvironment by comparing immune checkpoint activation in high and low TRIM22 expression groups. We assessed immune and stromal scores using the ESTIMATE R package, calculated immune infiltration status using TIMER (https://timer.cistrome.org/), and employed the Wilcoxon signed‐rank test to analyze immune cell differences.

Additionally, to understand the connection between prognostic models and the tumor immune microenvironment, we compared immune checkpoint activation in high and low expression groups. We also utilized ESTIMATE R package for immune and stromal scores, TIMER for immune infiltration status, and the Wilcoxon signed‐rank test for immune cell analysis.

### 2.13 Single‐cell analysis and functional state analysis

We utilized glioma single‐cell sequencing data (GSE57872) to investigate the relationship between SP140 and TRIM22 expression and the functional state of glioma cells. All data analyses and visualizations were conducted through Cancer SEA (http://biocc.hrbmu.edu.cn/CancerSEA/home.jsp) (Yuan et al., 2019).

### 2.14 Statistical analysis

Statistical analyses were conducted using GraphPad Prism 9.0 software. The results are presented as the mean ± standard deviation (SD). The normality of data distribution was assessed using the Bonferroni test. For datasets that exhibited a normal distribution, the Student's *t*‐test was employed for comparing two groups, whereas a one‐way ANOVA was used for comparisons involving multiple groups. Statistical significance was defined as *p* < .05. Significant findings were indicated with asterisks (*p < .05, **p < .01). Throughout the analysis, a 95% confidence level and consideration of effect sizes were taken into account.

## 3 RESULTS

### 3.1 Mutation status of the SP family genes in gliomas

**FIGURE 1:**
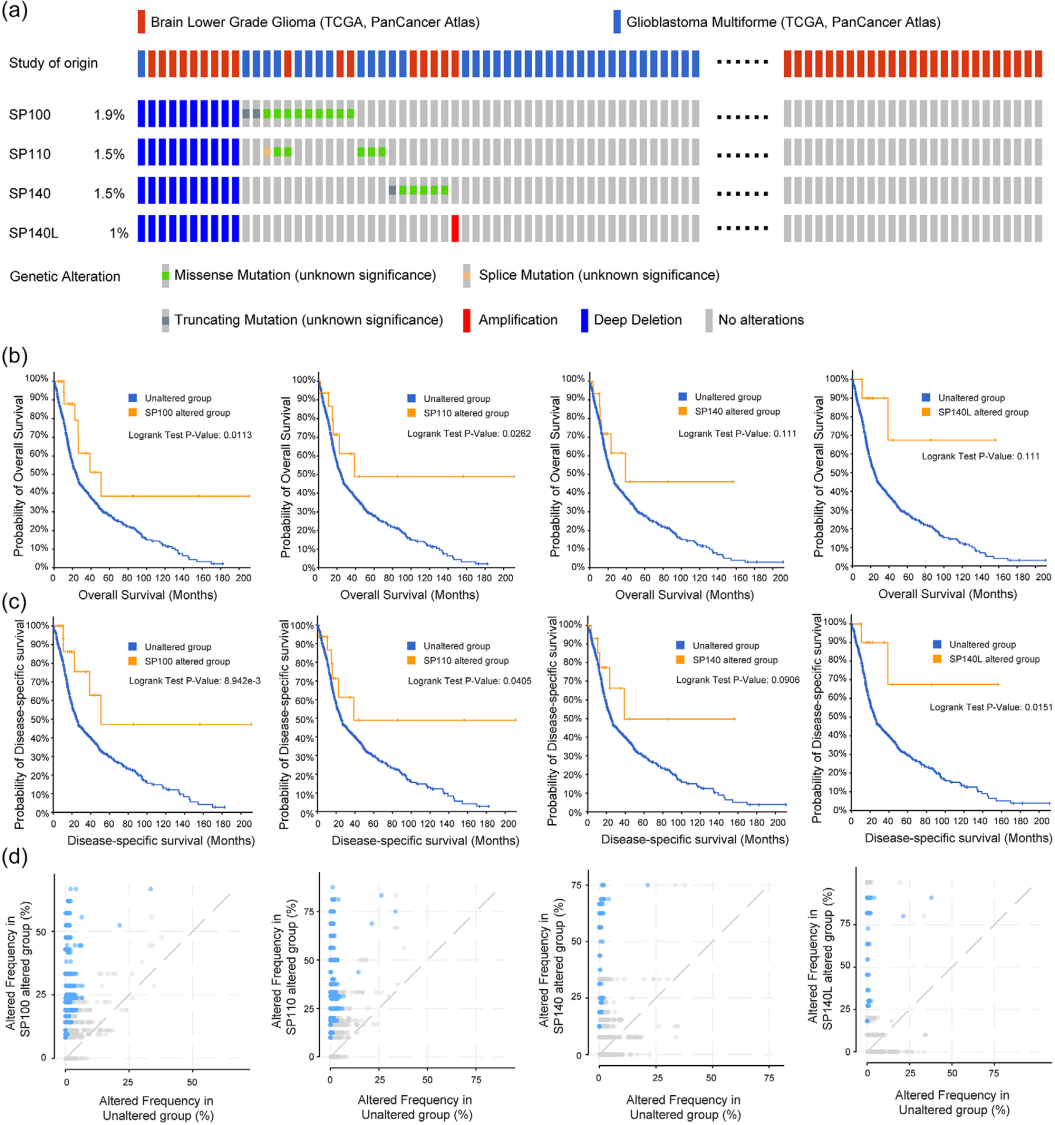
Genetic alterations and clinical significance of SP family in TCGA glioma cohort: (A) Rates and types of SP family genetic alterations in TCGA glioma dataset (*n* = 514 for LGG, 592 for GBM). Kaplan–Meier survival analysis for (B) overall survival (OS) and (C) disease‐specific survival (DSS) in glioma patients with and without SP family alterations. (D) Frequency of genomic alterations in samples with SP family gene alterations. Statistical significance was determined using log‐rank tests, with *p* < .05 considered significant.

Based on the mutation data of SP family genes and the TCGA genome, we generated a gene mutation waterfall plot for presentation. It is evident that all four SP family genes exhibit certain mutations in gliomas, with deep deletions being the most common mutation type, followed by missense mutations (Figure 1A).

We further investigated the impact of SP family mutations on survival rates using Kaplan–Meier curves. The results indicated that only specific gene mutations were associated with a poorer prognosis (Figure 1B and C). To determine whether these gene mutations directly contribute to the unfavorable prognosis associated with the SP gene family, we conducted an analysis of the mutation status of all genes in gliomas. We observed that mutations in the SP family genes tend to cooccur with mutations in other genes, and such cooccurring mutations are relatively infrequent (Figure 1D). Therefore, we do not attribute this phenomenon, linked to a worse prognosis, solely to gene mutations. Instead, we believe it is essential to focus more on gene expression levels.

### 3.2 Expression status of the SP family genes in gliomas

**FIGURE 2:**
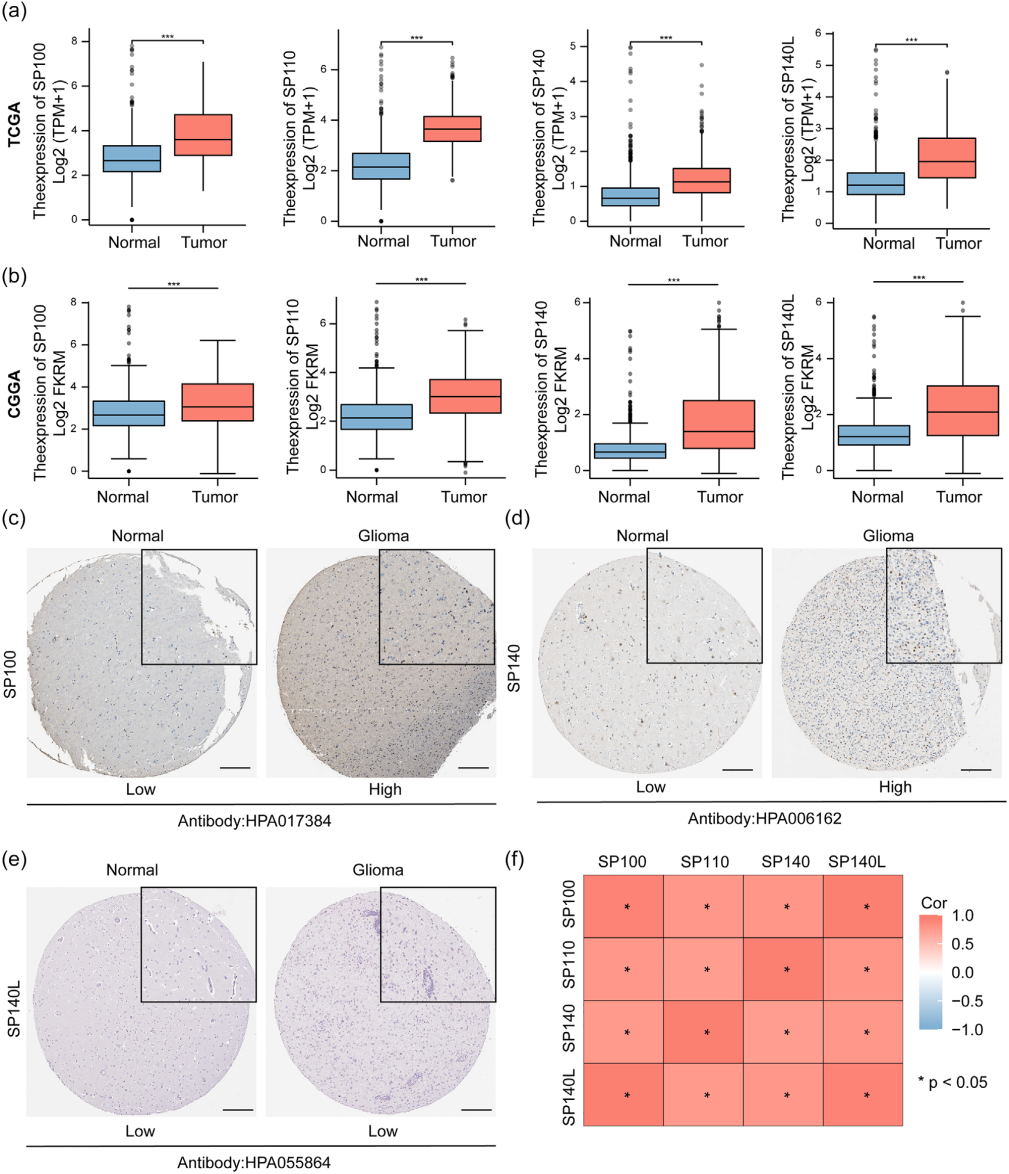
Differential expression of SP family mRNA and protein in glioma: (A) mRNA expression of SP Family in glioma and normal tissues from TCGA database. (B) mRNA expression of SP family in glioma and normal tissues from CGGA database. *p < .05, **p < .01, ***p < .005, ns indicates no significance. (C–E) Protein expression in each tissue, marked below the image. Scale bar: 4 µm, and the black square represents a 4× zoom. The antibody used for staining is indicated below. (F) Coexpression analysis of SP family in the TCGA database.

Upon comparing the expression levels of the SP family in glioma samples from the TCGA with normal brain tissue samples from the GTEx database, we discovered a general upregulation of the SP family in gliomas (Figure 2A). Similarly, when we compared glioma samples from the CGGA database to normal brain tissue samples from the GTEx database, we observed the same difference in expression levels (Figure 2B). These findings imply that the SP100 gene family might have a role in promoting the onset or progression of gliomas. To further confirm the alteration of SP family in the glioma tissue, we explored the protein levels of SP family in HPA. SP100 and SP140 showed a higher expression in the glioma tissue than in normal tissue (Figure 2C and D). However, SP140L showed a similar expression to normal tissue (Figure 2E). The expression of SP110 was missing in HPA. Overall, the expression of SP family was positively correlated with each other (Figure 2F).

### 3.3 Relationship between the expression levels of SP100 family genes and the prognosis of glioma patients

**FIGURE 3:**
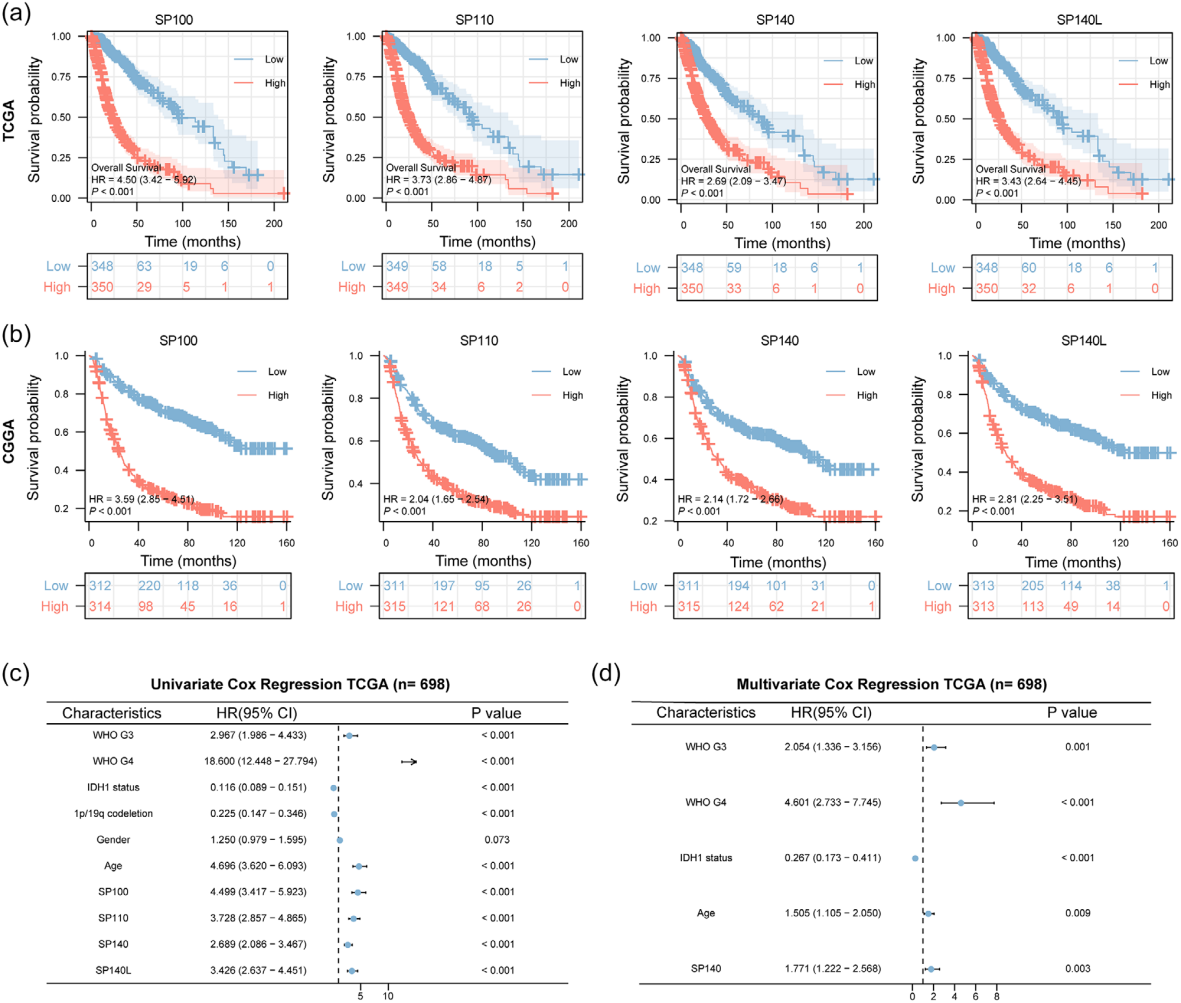
The prognostic value of SP family expression in glioma. (A) Kaplan–Meier survival analysis for overall survival in glioma patients from TCGA database stratified by SP family expression, *p*‐values obtained from log‐rank tests. (B) Kaplan–Meier survival analysis for overall survival in glioma patients from CGGA database stratified by SP family expression, *p*‐values obtained from log‐rank tests. (C, D) Forest plots displaying univariate and multivariate Cox proportional hazard ratios with 95% confidence intervals and *p*‐values for WHO grade, IDH1 status, 1p/19q codeletion, gender, age, and SP family in the TCGA database.

We employed Kaplan–Meier curves along with log‐rank tests to stratify glioma samples from the TCGA database into high and low expression groups, based on median gene expression levels. Subsequently, we generated survival curves for these two groups and calculated the associated differences. Our findings revealed that high expression of SP family correlated with a worse prognosis in glioma patients, with statistically significant distinctions observed between the high and low expression groups for each gene (*p* < .001) (Figure 3A).

Furthermore, we conducted a validation of this phenomenon using the CGGA database, and the results similarly indicated survival disparities linked to expression levels (Figure 3B). In the forest plot derived from univariate Cox regression analysis, all four genes within the SP family demonstrated statistical significance (Figure 3C). To pinpoint the most clinically meaningful gene among them, we incorporated additional clinical factors such as WHO grading, IDH1 mutation status, and age into a multivariate Cox regression analysis. The outcomes underscored the statistical significance of SP140 in relation to these clinical factors and its impact on prognosis (Figure 3D).

### 3.4 SP140 is an independent factor for predicting the prognosis of glioma patients

**FIGURE 4:**
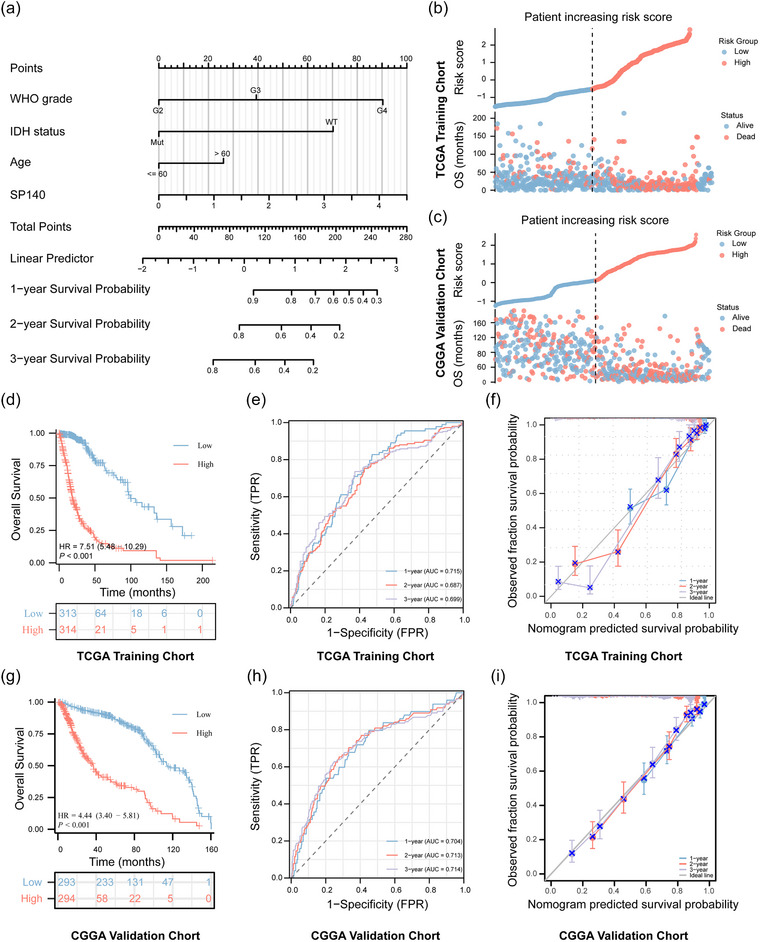
Construction of SP140‐based nomogram and risk score model for glioma patient survival prediction: (A) Nomogram incorporating independent prognostic clinicopathologic factors (SP140, WHO grade, IDH status, age) to predict 1‐, 2‐, and 3‐year survival probabilities for glioma patients. SP140‐based prognostic risk score model for risk stratification in (B) TCGA and (C) CGGA glioma patients, calculated based on independent prognostic clinicopathologic factors. (Kaplan–Meier survival analysis for overall survival in (D) TCGA and (G) CGGA glioma patients, categorized into low‐ and high‐risk groups based on median risk scores, with *p*‐values from log‐rank tests. Time‐dependent receiver operator characteristic curve (t‐ROC) assessing predictive accuracy of SP140‐based prognostic risk score model in glioma patients from (E) TCGA and (H) CGGA databases, calculated for 1‐, 2‐, and 3‐year AUCs. Calibration curves evaluating consistency between predictive and observed probabilities of the SP140‐based prognostic risk score model in glioma patients from (F) TCGA and (I) CGGA databases, assessed by comparing ideal and predictive lines for 1‐, 2‐, and 3‐year predictions.

To further investigate the relationship between SP140 and the prognosis of glioma patients, we conducted a nomogram analysis. This analysis incorporated clinical factors, including WHO grading, IDH mutation status, age, and SP140 expression, to predict 1‐year, 2‐year, and 3‐year survival rates following glioma diagnosis. Each variable was assigned a weighted score on a scale from 1 to 100 based on the results of the multivariable Cox regression analysis, as illustrated in the nomogram point system (Figure 4A). By drawing a line from the total score line to the survival outcome line, the nomogram provides the estimated survival probability for each glioma patient at 1 year, 2 years, and 3 years.

Additionally, we further validated the prognostic value of clinical information and SP140 expression in these patients. We selected these variables and, through multivariate Cox analysis, computed a risk score for each glioma patient by combining the coefficients of each variable, thus creating a prognostic risk score model (Figure 4B).

Initially, we employed a cohort of glioma patients (*n* = 100) from the TCGA database as a training set. We divided the TCGA glioma samples into high‐risk and low‐risk score groups based on the calculated risk scores. Kaplan–Meier curves demonstrated that patients in the high‐risk score group had a worse prognosis (Figure 4D). To validate the accuracy of the risk score model, we analyzed time‐dependent receiver operating characteristic (t‐ROC) curves and calculated the area under the curve (AUC) as the curve evolved over time (Figure 4E). Calibration curve analysis based on this model was also performed (Figure 4F). The results consistently revealed AUC values exceeding 0.6 at 1‐year, 2‐year, and 3‐year time points, indicating that the model consistently exhibited the best agreement between predicted and observed survival rates during these time intervals. This suggests that the prognostic risk score model, incorporating SP140 expression and clinical information, is accurate and reliable.

Furthermore, we applied the same modeling approach to glioma samples (*n* = 100) from the CGGA database for external validation (Figure 4C, G, H, and I). The results confirmed that this modeling method was equally applicable to glioma samples from the CGGA database, underscoring the model's robustness.

### 3.5 The biological roles and downstream gene of SP140

**FIGURE 5:**
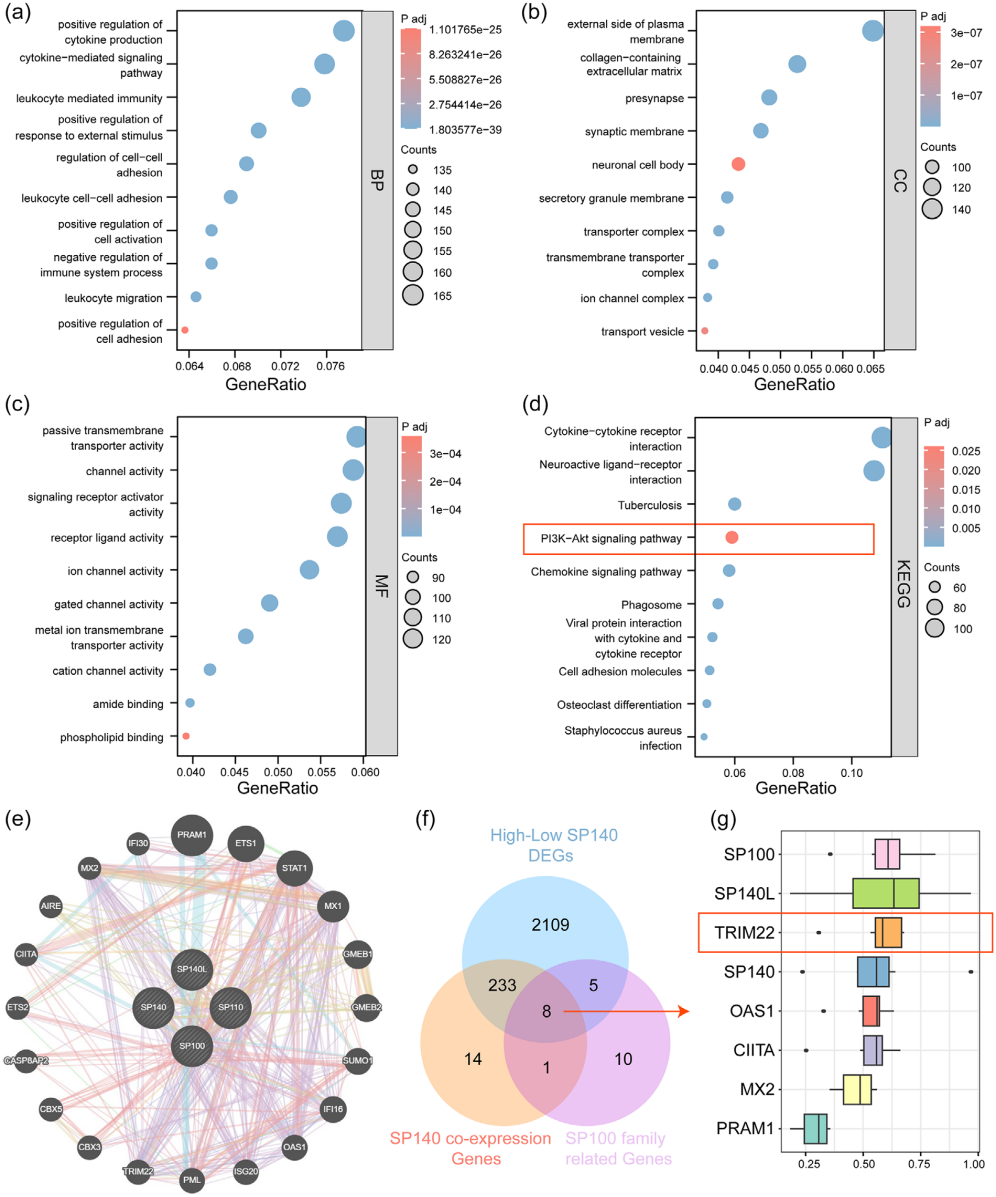
The biological roles and downstream gene of SP140. GO (A) biological process (BP), (B) cellular components (CC), (C) molecular functions (MF), and (D) the KEGG pathways influenced by DEGs between low and high expression of SP140 groups. (E) Genemania showed 20 proteins with the highest interaction strength in the SP family. (F) Venn diagram showed the intersected genes. (G) Friends analysis showed the functional correlations among different genes within a pathway. The red box emphasized that TRIM22 exhibited the strongest correlation with other genes.

To further explore the oncogenic roles of SP140 in glioma, we differential expression analysis on mRNA transcripts of all coding proteins between the high and low expression of SP140 groups. According to the differentially expressed genes, GO and KEGG analysis were performed to explore the biological functions. GO biological progress (BP), cellular component (CC), and molecular function (MF) showed the DEGs between the high and low expression of SP140 groups were associated with immune and signaling pathway regulation (Figure 5A–C). KEGG analysis showed that the DEGs were closely related to PI3K/AKT signaling pathway (Figure 5D). In order to explore the downstream gene of SP140, we screened out 20 SP family related genes via GENEMINIA (Figure 5E). Then, we intersected differentially expressed genes in the SP140 high and low expression groups, coexpressed genes with SP140, and interacting genes within the SP family, resulting in a total of 8 candidate genes of interest (Figure 5F). Upon conducting Friends analysis on these 8 genes, the results revealed that TRIM22 exhibited the strongest correlation with other genes (Figure 5G). Previous studies have also indicated that TRIM22 can influence cell proliferation by modulating the PI3K/AKT pathway (Li et al., 2018; Ren et al., 2022), which aligns with our findings.

### 3.6 SP140 inhibitor suppress glioma progress and PI3K/AKT signaling pathway

**FIGURE 6:**
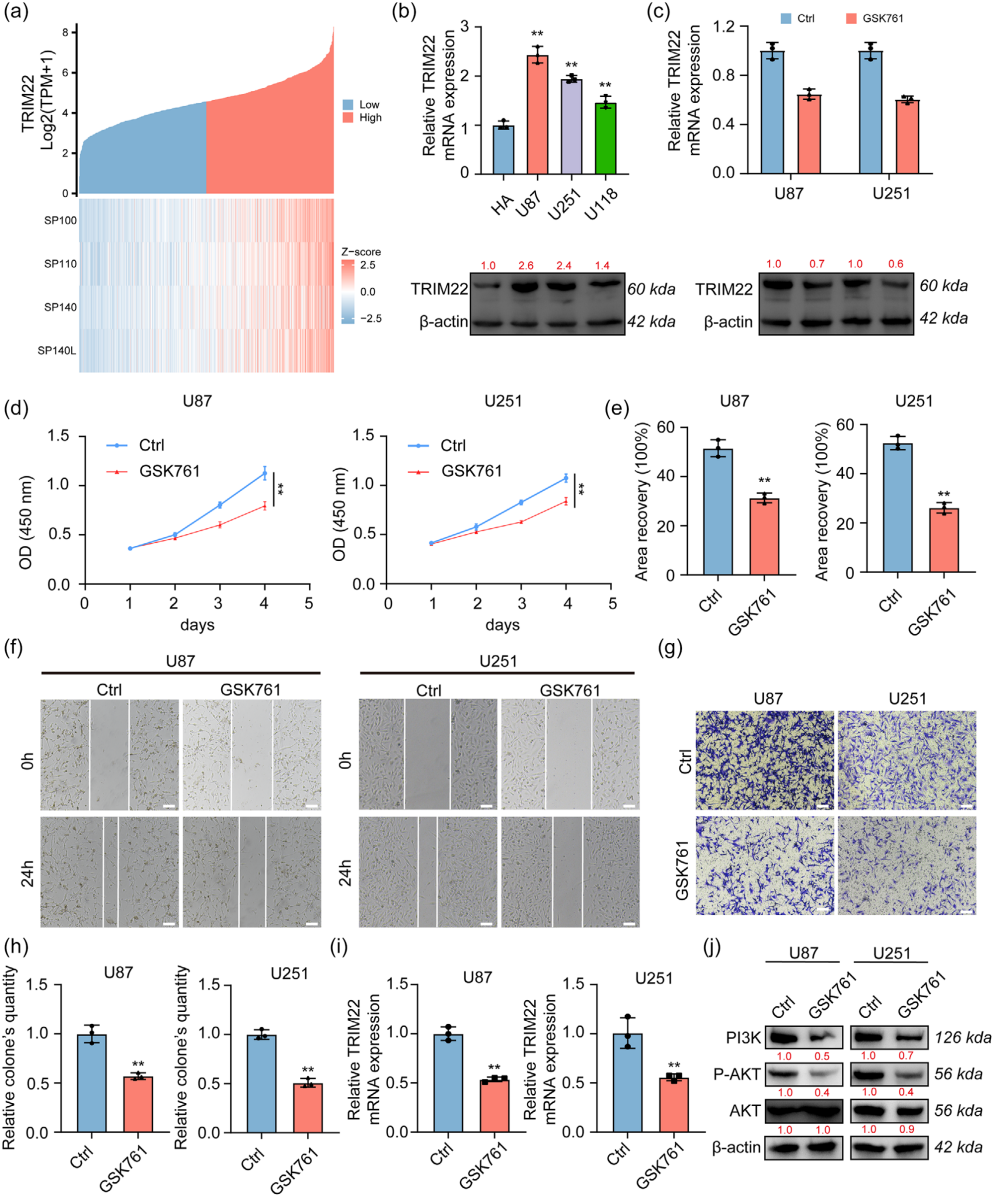
SP140 inhibitor suppress glioma progress and TRIM22/PI3K/AKT signaling pathway. (A) Coexpression analysis of TRIM22 and SP family in TCGA glioma database. (B) The mRNA and protein expression level of TRIM22 in different cell lines. (C) The mRNA and protein expression level of TRIM22 in U87 and U251 after GSK761 intervention. (D) CCK‐8 analysis showed cell proliferation in U87 and U251 after GSK761 intervention. (E, F) Wound‐healing assay showed cell migration in U87 and U251 after GSK761 intervention. (G, H) Transwell invasion assay showed cell invasion in U87 and U251 after GSK761 intervention. (I) The mRNA expression level of TRIM22 after GSK761 treatment. (J) Western Blot analysis showed PI3K/AKT signaling pathway changes after GSK761 intervention. *p < .05, **p < .01, ns indicates no significance.

The coexpression analysis reveals a positive correlation between the expression of TRIM22 and four different SP family genes (Figure 6A). We examined the expression levels of the mRNA and protein in human astrocytes (HA) and three GBM cell lines. The results showed that TRIM22 was significantly higher expressed in GBM cell lines, especially in U87 and U251 than HA (Figure 6B). After intervention with the SP140 inhibitor GSK761, there was a significant decrease in both the mRNA and protein levels of TRIM22 (Figure 6C). GSK761 also significantly inhibited the proliferation of U87 and U251 cell lines (Figure 6D). Wound‐healing and transwell assay showed that GSK761 significantly suppressed the invasion and migration of U87 and U251 cells (Figure 6E–H). To validate whether GSK761 can regulate glioma progression through the PI3K/AKT pathway, we conducted Western Blot analysis to examine the levels of proteins associated with the pathway. The results demonstrated that GSK761 significantly downregulated the mRNA level of TRIM22 (Figure 6I) and the levels of PI3K and phosphorylated AKT (Figure 6J), indicating that GSK761 may inhibit the PI3K/AKT signaling pathway.

### 3.7 Signal‐cell analysis and TRIM22 mediated glioma immune infiltration

**FIGURE 7:**
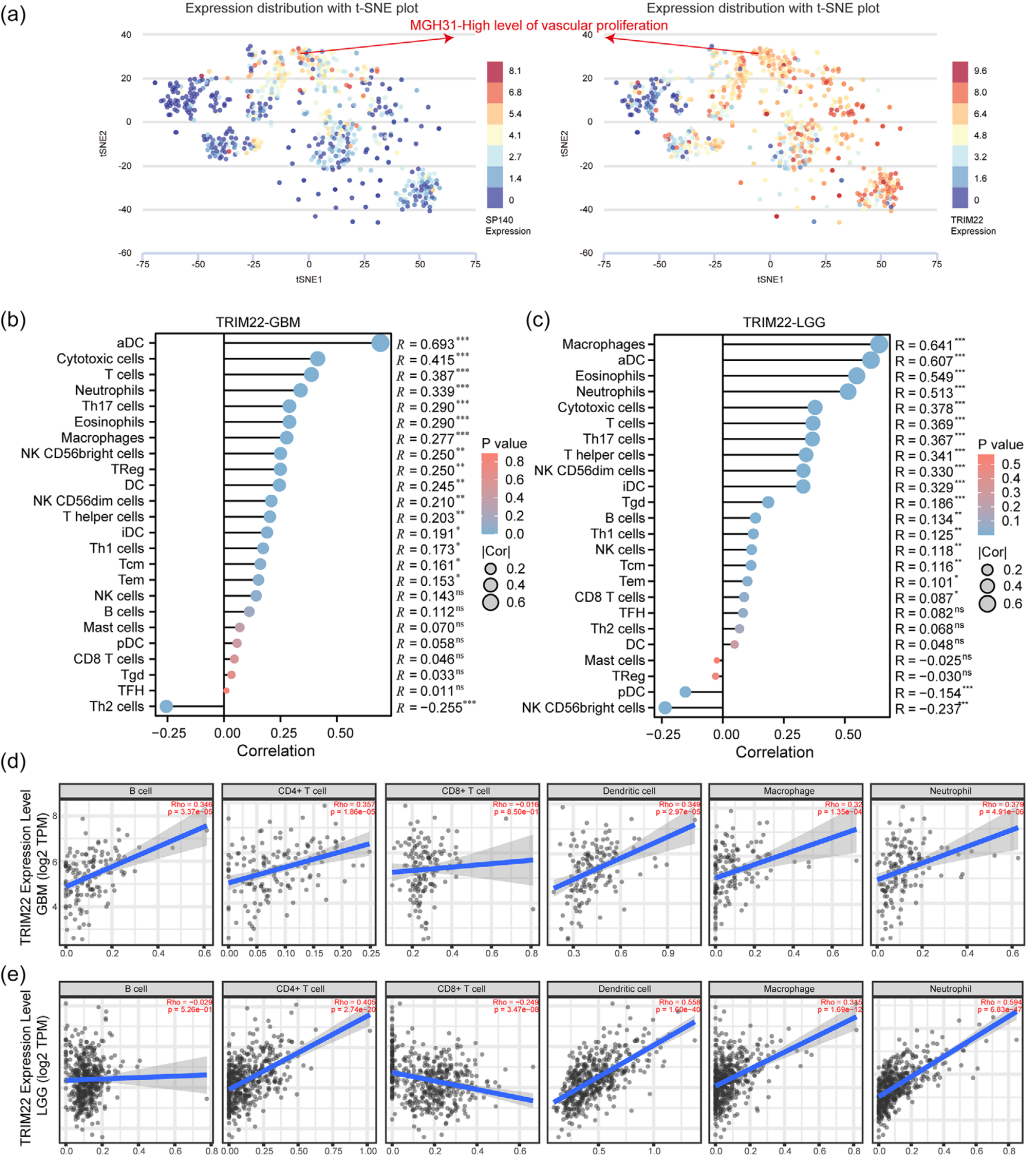
Signal‐cell analysis and TRIM22 mediated glioma immune infiltration. (A) Differential expression distribution of SP140 and TRIM22 in glioma samples using single‐cell analysis. Relationship between TRIM22 expression and infiltration levels of various immune cells in TCGA (B) GBM and (C) LGG databases. Relationship between TRIM22 mRNA expression and infiltration levels of various immune cells in TCGA (D) GBM and (E) LGG databases.

To investigate the impact of SP140 and TRIM22 expression on the functional status of glioma, we proceeded with single‐cell analysis using the primary glioma dataset (GSE57872) and employed CancerSEA (http://biocc.hrbmu.edu.cn/CancerSEA/home.jsp) for the analysis and visualization of the results. The single‐cell analysis results reveal that coexpression of SP140 and TRIM22 occurs in a subset of cells exhibiting high level of vascular proliferation (Figure 7A). To validate the correlation between TRIM22 and immune cell infiltration, we conducted an analysis of immune cells associated with TRIM22. The results indicate a positive correlation between TRIM22 and various immune cell types, both in GBM and LGG (Figure 7B and C), suggesting that the impact of TRIM22 on immune cell infiltration is extensive. We conducted an immune infiltration analysis using the TIMER method on glioma samples from TCGA to explore the relationship between immune infiltration and TRIM22. The results revealed that, with the exception of negative correlations between TRIM22 and immune infiltration in B cells and CD8+ T cells in LGG (Figure 7D and E), TRIM22 exhibited significant positive correlations with CD4+ T cells, dendritic cells, macrophages, and neutrophils in both LGG and GBM. Considering the well‐documented positive impact of immune cell therapy on the clinical outcomes of gliomas (Sokratous et al., 2017), these findings suggest that TRIM22 may serve as a promising indicator for assessing the potential efficacy of immunotherapy in glioma treatment.

## 4 DISCUSSION

The SP family, known for its multifaceted roles in health and disease, encompassing gene regulation, immune responses, and DNA interactions, has indeed been implicated in various aspects of tumor initiation, progression, and recurrence (Sokratous et al., 2017). However, the clinical significance and practical applications of the SP family in the context of glioma remain incompletely understood. In this study, we embarked on a comprehensive analysis of SP family expression and its prognostic implications in gliomas, utilizing data from the TCGA and CGGA databases. Our research findings reveal that the SP family displays heightened expression and genetic alterations in gliomas, affecting prognosis. However, when subjected to regression analysis accounting for multiple factors involving clinical characteristics, only SP140 emerged as an independent predictor of glioma prognosis. Consequently, we constructed and validated a novel SP140‐based nomogram for predicting the survival of patients with glioma. Furthermore, we investigated the mechanisms by which SP140 influences glioma, revealing that it may regulate glioma proliferation and invasion through the TRIM22‐mediated modulation of the PI3K/AKT signaling pathway. These findings shed light on the potential therapeutic targets for addressing this challenging disease.

Mutations in the SP family of genes can potentially impact the prognosis of gliomas. However, these mutations may be correlated with alterations in other genes, thus not necessarily emerging as independent prognostic factors. In terms of mRNA expression levels, SP140 but not other members of SP family could be identified as an independent predictor of glioma prognosis although involving clinical characteristics. This may be attributed to its more critical role in the development and progression of gliomas, making its influence less susceptible to interference or correction by other factors. Furthermore, the mechanism of action of SP140 may involve specific signaling pathways or biological processes, rendering it an independent predictive factor.

To further investigate the mechanism by which SP140 regulates glioma progression, we conducted a screening of SP140‐related genes and identified TRIM22 as having a high pathway correlation via Friends analysis. TRIM22 (Tripartite Motif Containing 22) belongs to the TRIM family (Tripartite Motif) and is primarily localized within the cell nucleus (Gao et al., 2009). The protein encoded by TRIM22 is an E3 ubiquitin ligase, and it plays a role in various biological processes within the cell, including immune responses, apoptosis, antiviral defense, and cell signaling (Duan et al., 2008). In gliomas, TRIM22 is believed to be closely associated with the NF‐KB and MAPK pathways, regulating glioma proliferation and invasion (Fei et al., 2023; Ji et al., 2021). In other types of tumors, TRIM22 has also been linked to various signaling pathways. For example, in osteosarcoma, TRIM22 can impact autophagy by modulating the ROS/AMPK/mTOR signaling pathway (Liu et al., 2022), while in lung cancer, it can influence epithelial‐mesenchymal transition through the AKT/GSK3β signaling pathway (Liu et al., 2022). Besides, TRIM22 knockdown inhibited the activation of PI3K/Akt/mTOR pathway by decreasing the level of the phosphorylated form p‐Akt and p‐mTOR in chronic myeloid leukemia (Li et al., 2018). However, no previous studies have investigated whether SP140 was associated with the PI3K/AKT pathway. Our research findings indicated that in gliomas, SP140 inhibitors could suppress the PI3K/AKT signaling pathway by downregulating TRIM22, thus inhibiting glioma proliferation and invasion. As a chromatin reader, how SP140 regulates the expression of TRIM22 and whether it can directly modulate the PI3K/AKT signaling pathway remain unknown. Further research is needed to delve into the intricate mechanisms by which SP140 regulates downstream proteins.

Single‐cell analysis revealed that SP140 and TRIM22 were coexpressed at high levels in a subset of cells exhibiting elevated angiogenic activity (Patel et al., 2014). Previous studies confirmed that high angiogenic activity was closely related to PI3K/AKT signaling pathway (Park et al., 2017; Zhang et al., 2016; Zhu et al., 2018), which further substantiated our findings. In addition, we conducted an analysis of immune infiltration for TRIM22 and observed a positive correlation between TRIM22 and various immune cell infiltrations. These findings suggested that SP140 might regulate the immune system through TRIM22/PI3K/AKT axis. However, further experimental studies would be needed to confirm this regulatory mechanism conclusively.

Despite the practicality of our findings, there are certain limitations in our study that should be acknowledged and addressed. First, even though the other members of SP family may not act as independent factors, they could potentially exert some level of influence on gliomas, warranting further exploration. Second, while we included two large glioma cohorts in our study, the prognostic model could be further refined and validated in even larger datasets to enhance its robustness in future research. Third, our validation of human samples was limited to the HPA database and cell lines U87 and U251. More attention should be given to validation in xenograft models and human samples. Fourth, while we demonstrated in our paper that SP140 affects the PI3K/AKT signaling pathway through TRIM22, we cannot rule out the possibility that SP140 may also impact other signaling pathways. Finally, the effects of SP140 and TRIM22 on immune infiltration should be further studied in in vivo models. Anyway, our research focus on SP family may lead to valuable insights and potential therapeutic targets for patients undergoing glioma.

## AUTHOR CONTRIBUTIONS

**Xiang Li**: Supervision; resources; writing—original draft; writing—review and editing; software. **Guangzhao Li**: Investigation; methodology; software; writing—original draft; validation; resources. **Longyuan Li**: Writing—original draft; supervision; methodology; visualization; validation. **Bixi Gao**: Methodology; validation; writing—original draft; formal analysis; resources; data curation; software. **Xiaowang Niu**: Validation; software. **Yunjiang Wang**: Writing—review and editing; conceptualization; funding acquisition; project administration. **Zhong Wang**: Project administration; conceptualization; writing—review and editing.

## CONFLICT OF INTEREST STATEMENT

The authors declare that the research was conducted in the absence of any commercial or financial relationships that could be construed as a potential conflict of interest.

## FUNDING INFORMATION

Our research has not received any funding support.

### PEER REVIEW

The peer review history for this article is available at https://publons.com/publon/10.1002/brb3.3465.

## Data Availability

The datasets presented in this study can be found in online repositories.
